# Information about the new coronavirus disease (COVID-19)

**DOI:** 10.1590/0100-3984.2020.53.2e1

**Published:** 2020

**Authors:** Claudio Márcio Amaral de Oliveira Lima

**Affiliations:** 1 Radiologist for United Health Group Inc. (UHG), Rede D’Or São Luiz and for Rede Casa, Rio de Janeiro, RJ, Brazil. Email: cmaolima@gmail.com.

Coronavirus is a zoonotic virus, an RNA virus in the family Coronaviridae of the order Nidovirales^(1)^. It is a family of viruses that cause respiratory infections, which were first isolated in 1937 and designated coronaviruses, because they have a crown-like appearance under microscopy, in 1965^(2)^. The types of coronavirus known to date are as follows: the alpha coronaviruses HCoV-229E and HCoV-NL63; the beta coronaviruses HCoV-OC43 and HCoV-HKU1; SARS-CoV, which causes severe acute respiratory syndrome (SARS); MERS-CoV, which causes Middle East respiratory syndrome (MERS); and SARS-CoV-2, a new coronavirus described in late 2019 after cases were reported in China^(2)^, which causes the disease known as coronavirus disease 2019 (COVID-19).

The definitive diagnosis of COVID-19 is made by analyzing respiratory samples (collected by aspiration of the airways or sputum induction). Laboratory tests to identify the virus involve the use of real-time polymerase chain reaction techniques and partial or total sequencing of the viral genome. The collection of nasopharyngeal aspirate, combined (nasal and oral) swab samples, or samples of lower respiratory tract secretions (sputum, tracheal lavage fluid, or bronchoalveolar lavage fluid) is recommended. To confirm the disease, it is necessary to perform molecular biology tests that detect viral RNA. Severe cases should be transferred to a referral hospital for isolation and treatment. Individuals with mild symptoms should be followed at the primary health care level and should be advised to selfisolate at home^(1)^.

The clinical spectrum of coronavirus infection is quite broad, ranging from a simple cold to severe pneumonia. Clinically, COVID-19 initially presents as a flu-like syndrome. Individuals with COVID-19 usually develop signs and symptoms, such as mild respiratory illness and persistent fever, an average of 5-6 days after infection (range, 1-14 days). The fever is persistent, in contrast with the progressive decline observed in cases of influenza^(1,3)^. Fever may not be present in some cases, such as those occurring in patients that are very young, elderly, or immunocompromised, as well as in those that have used antipyretic medication^(1)^. In children, the disease appears to be relatively rare and mild, only approximately 2.4% of all reported cases occurring in individuals under 19 years of age, of whom only 2.5% and 0.2% have developed severe or critical illness, respectively^(1,3)^.

According to the Clinical Management Protocol for the New Coronavirus, disseminated by the Brazilian National Ministry of Health in February of this year^(1)^, males and individuals over 50 years of age predominated among the first 99 patients hospitalized with pneumonia and a confirmed diagnosis of COVID-19 at a hospital in the city of Wuhan. Among those 99 patients, the main symptoms were fever (in 83%), cough (in 82%), shortness of breath (in 31%), muscle pain (in 11%), mental confusion (in 9%), headache (in 8%), sore throat (in 5%), rhinorrhea (in 4%), chest pain (in 2%), diarrhea (in 2%), and nausea/vomiting (in 1%). In another study, involving 41 patients diagnosed with COVID-19, lymphopenia was reported.

Evaluating data from 1,099 patients with confirmed COVID-19 in China, Guan et al.^(4)^ observed that the mean age of the patients was 47 years and that 41.9% of the patients were female. The primary composite outcome-defined as admission to the intensive care unit (ICU), the need for artificial ventilation, or progression to death-occurred in 67 patients (6.1%), admission to the ICU occurring in 5.0%, invasive mechanical ventilation being required in 2.3%, and death occurring in 1.4%. The most common symptoms were fever (in 43.8% at admission and 88.7% during hospitalization) and cough (in 67.8%). Diarrhea occurred in only 3.8% of cases. Lymphopenia was present in 83.2% of patients on admission. The mean incubation period was 4 days. Patients often presented without fever and many had normal X-ray findings.

Based on a study of 55,924 confirmed cases, the World Health Organization-China Joint Mission on Coronavirus Disease 2019^(3)^ reported the most common signs and symptoms: fever (in 87.9%); dry cough (in 67.7%); fatigue (in 38.1%); sputum production (in 33.4%); dyspnea (in 18.6%); sore throat (in 13.9%); headache (in 13.6%); myalgia or arthralgia (in 14.8%); chills (in 11.4%); nausea or vomiting (in 5%); nasal congestion (in 4.8%); diarrhea (in 3.7%); hemoptysis (in 0.9%); and conjunctival congestion (in 0.8%). In most cases, the disease was mild and there was complete recovery. Among patients with laboratory-confirmed COVID-19, approximately 80% had mild to moderate disease, which includes cases with and without pneumonia; 13.8% had severe disease, which includes dyspnea, respiratory rate ≥ 30 breaths/min, peripheral oxygen saturation ≤ 93%, and arterial oxygen tension/fraction of inspired oxygen ratio < 300, with or without pulmonary infiltrate occupying more than 50% of the lung parenchyma in the first 24-48 h; and 6.1% had critical disease, which includes respiratory failure and septic shock, with or without multiple organ dysfunction/failure. Although asymptomatic infection has been reported, the proportion of truly asymptomatic cases is not well defined. The individuals most at risk of serious illness and death reportedly include people over 60 years of age, especially those with underlying conditions, such as hypertension, diabetes, cardiovascular disease, chronic respiratory disease, and cancer^(1,3)^.

In patients with COVID-19, the CT findings most commonly reported are ground-glass opacities and areas of consolidation, sometimes with a rounded morphology and peripheral distribution. Bernheim et al.^(5)^ evaluated pulmonary abnormalities related to the duration of the disease and reported that, on chest CT, the disease was most extensive at approximately 10 days after symptom onset. A chest X-ray is often essential for the evaluation of patients with suspected COVID-19. Immediate recognition of the disease is essential to ensure timely treatment. From a public health point of view, rapid isolation of the patient is crucial to containing this communicable disease.

In the Bernheim et al. study^(5)^, the chest CT findings of 121 patients infected with COVID-19 in China were characterized in relation to the time from the onset of symptoms to the first CT scan. The authors hypothesized that the frequency of certain CT findings would increase in parallel with an increase in the time elapsed since infection. Only the initial chest CT scans were evaluated. In 27 (22%) of the 121 patients, CT showed no changes. The remaining 94 (78%) patients had ground-glass opacities, areas of consolidation, or both. Of the 121 patients, 73 (60%) had bilateral lung disease. None of the 121 patients had thoracic lymph node disease, pulmonary cavitation, or pulmonary nodules, and only 1 patient (1%) had pleural effusion. The time from the onset of symptoms to the first CT scan was classified as early (0-2 days; 36 patients), intermediate (3-5 days; 33 patients), or late (6-12 days; 25 patients). The frequency of ground-glass opacities and areas of consolidation was considerably lower in the early-CT group than in the intermediate-CT group and late-CT group. Pulmonary opacities were seen in only 16 (44%) of the 36 early-CT group patients, compared with 30 (91%) of the 33 intermediate-CT group patients and 24 (96%) of the 25 late-CT group patients. Bilateral pulmonary involvement was observed in 10 (28%) of the early-CT group patients, in 25 (76%) of the intermediate-CT group patients, and in 22 (88%) of the late-CT group patients. Linear opacities, a crazy-paving pattern, and the inverted halo sign were absent in the early-CT group but were present in the late-CT group in 5 (20%), 5 (20%), and 1 (4%) of patients, respectively. In terms of disease distribution in the axial plane, peripheral distribution was observed in 8 (22%) of the early-CT group patients, in 21 (64%) of the intermediate-CT group patients, and in 18 (72%) of the late-CT group patients.

The recognition of imaging patterns based on the time since coronavirus infection is critical not only to understanding the pathophysiology and natural history of the infection but also to predicting patient progression and the development of complications. So far, the anatomopathological aspects of the disease have not been described^(6)^. Future studies will be able to evaluate imaging findings in patients with chronic COVID-19(^5^), as well as to report the pathological aspects of the infection.

## References

[1] Brasil, Ministério da Saúde Protocolo de manejo clínico para o novo-coronavírus (2019-nCoV).

[2] Brasil, Ministério da Saúde Coronavírus: o que você precisa saber e como prevenir o contágio.

[3] Report of the WHO-China Joint Mission on Coronavirus Disease 2019 (COVID-19).

[4] Guan W, Ni Z, Hu Y (2020). Clinical characteristics of coronavirus disease 2019 in China. N Engl J Med.

[5] Bernheim A, Mei X, Huang M (2020). Chest CT findings in coronavirus disease-19 (COVID-19): relationship to duration of infection. Radiology.

[6] Kanne JP, Little BP, Chung JH (2020). Essentials for radiologists on COVID-19: an update-Radiology Scientific Expert Panel. Radiology.

